# Identification and Validation of a Nine-Gene Amino Acid Metabolism-Related Risk Signature in HCC

**DOI:** 10.3389/fcell.2021.731790

**Published:** 2021-09-07

**Authors:** Yajuan Zhao, Junli Zhang, Shuhan Wang, Qianqian Jiang, Keshu Xu

**Affiliations:** Division of Gastroenterology, Union Hospital, Tongji Medical College, Huazhong University of Science and Technology, Wuhan, China

**Keywords:** amino acid metabolism, gene, prognosis, immune infiltration, nomogram, hepatocellular carcinoma

## Abstract

**Background:** Hepatocellular carcinoma (HCC) is the world’s second most deadly cancer, and metabolic reprogramming is its distinguishing feature. Among metabolite profiling, variation in amino acid metabolism supports tumor proliferation and metastasis to the most extent, yet a systematic study on the role of amino acid metabolism-related genes in HCC is still lacking. An effective amino acid metabolism-related prediction signature is urgently needed to assess the prognosis of HCC patients for individualized treatment.

**Materials and Methods:** RNA-seq data of HCC from the TCGA-LIHC and GSE14520 (GPL3921) datasets were defined as the training set and validation set, respectively. Amino acid metabolic genes were extracted from the Molecular Signature Database. Univariate Cox and LASSO regression analyses were performed to build a predictive risk signature. K-M curves, ROC curves, and univariate and multivariate Cox regression were conducted to evaluate the predictive value of this risk signature. Functional enrichment was analyzed by GSEA and CIBERSORTx software.

**Results:** A nine-gene amino acid metabolism-related risk signature including B3GAT3, B4GALT2, CYB5R3, GNPDA1, GOT2, HEXB, HMGCS2, PLOD2, and SEPHS1 was constructed to predict the overall survival (OS) of HCC patients. Patients were separated into high-risk and low-risk groups based on risk scores and low-risk patients had lower risk scores and longer survival time. Univariate and multivariate Cox regression verified that this signature was an independent risk factor for HCC. ROC curves showed that this risk signature can effectively predict the 1-, 2-, 3- and 5-year survival times of patients with HCC. Additionally, prognostic nomograms were established based on the training set and validation set. These genes were closely correlated with the immune regulation.

**Conclusion:** Our study identified a nine-gene amino acid metabolism-related risk signature and built predictive nomograms for OS in HCC. These findings will help us to personalize the treatment of liver cancer patients.

## Introduction

A series of biochemical changes during cancer development can promote infinite tumor cell proliferation, activate tissue invasion and metastasis, and prevent tumor cell growth from being inhibited. Metabolic reprogramming is one of the most critical biochemical variations observed in cancer (Boroughs and DeBerardinis, 2015). Amino acids are essential nutrients and energy sources for tumor cells. Amino acids associate with the metabolism of glucose, lipids and nucleotides, which are crucial for tumor proliferation, invasion and metastasis (Li and Zhang, 2016; Vettore et al., 2020). Many cancers require exogenous supplementation with glutamine to maintain tumor cell proliferation, in a process called “glutamine dependence”(Lukey et al., 2017). Serine, glycine and threonine metabolism and the one-carbon unit product derived from these processes properly satisfy tumor cell proliferation and maintain the redox, genetic and epigenetic state (Locasale, 2013).

Increasing studies have supported that amino acid metabolic genes are vitally important in tumor development. Glutaminase 2 (GLS2) encodes glutaminase, which can induce tumor cells to resist ROS-related apoptosis and enhance drug resistance through p53 mediated transcription (Matés et al., 2020). Serine hydroxymethyltransferase 2 (SHMT2) can be induced by both c-Myc and HIF1α to enhance the ability to resist hypoxia-induced tumor cell death and promote the invasion of various cancers (Ye et al., 2014). Solute carrier family 7 member 8 (SLC7A8) is an important branched-chain amino acid (BCAA) transporter. SLC7A8 regulates the activation of glutamine-dependent mTOR and enhances the resistance of pancreatic cancer to gemcitabine, which promotes tumor proliferation and inhibits apoptosis (Feng et al., 2018).

Globally, liver cancer is the second most deadly tumor, with a 5-year survival rate of 18% (Villanueva, 2019). Hepatocellular carcinoma is the most common type of liver cancer, accounting for 90% (Forner et al., 2018). Current treatments such as surgical therapy, chemotherapy, and radiotherapy have significantly suppressed cancer proliferation and improved the survival of HCC patients. However, HCC displays a high degree of molecular heterogeneity among patients, at different locations within a patient, and even within a single tumor, which is closely correlated with the common occurrence of drug resistance and relapse after surgical resection and comprehensive treatment, consequently leading to a poor prognosis (Li and Wang, 2016; Xu et al., 2019). It is urgent that more effective biomarkers be identified to provide individual treatment for HCC patients. The liver is the central organ for amino acid metabolism and the importance of amino acid metabolism in HCC has been noticed in recent years. Aberrant amino acid and protein metabolism provided active biosynthesis support for HCC. For example, branched-chain amino acid (BCAA) metabolism disorders are common in HCC, such as upregulation of isoleucine and downregulation of glutamate (Di Poto et al., 2017). Moreover, an increasing proportion of BCAAs restrains the proliferation of HepG2 liver tumor cells and helps to recover liver functions and prevent early recrudescence after surgical resection (Tajiri and Shimizu, 2013). A recent study revealed that BCAA reduction is an independent risk factor for sarcopenia in the course of HCC recurrence, worsening the prognosis of HCC patients (Sano et al., 2021). Research on amino acid metabolism is of great significance for the prevention and treatment of HCC. HCC tumor cell can active immune reaction and provide appropriate tumor microenvironment for cancer development (Long et al., 2019). B and T cells played a vital role in the HCC tumor microenvironment. NK cell decreasing enrolled in HBV or HCV infection and promote the progression of liver cancer (Sun et al., 2015). However, whether amino acid metabolism-related genes are involved in immune regulation of HCC remains unclear. Moreover, effective amino acid metabolic genes to predict the overall survival (OS) of patients with HCC are still lacking.

In this study, we obtained HCC RNA sequencing data from the TCGA database and the GEO database, mined amino acid metabolism-related genes closely related to the OS of HCC patients, and then established an effective signature of amino acid metabolism-related biomarkers to extend the knowledge of molecular mechanisms and clinical prognosis of HCC.

## Materials and Methods

### Data Collection

We downloaded the RNA-seq (HTseq-FPKM) data and clinical data of HCC from the TCGA database.^1^ The TCGA barcode was used to match different patients, and 370 HCC and 50 normal samples were selected as the training set. The clinical information included age, gender, histological grade, stage, TNM classification, survival time and survival status.

The GSE14520 (GPL3921) dataset was obtained from the GEO database, which contained gene expression and clinical data of HCC, paired non-tumor tissues, and healthy liver tissues analyzed by Affymetrix microarray profiling. Finally, 221 tumor samples were selected as the validation set. The clinical information included age, gender, main tumor size, number of tumors, TNM staging, BCLC staging and CLIP staging. Clinical data of the two cohorts are listed in Table 1.

**TABLE 1:** The clinicopathological characteristics of HCC patients in the training set and validation set.

Parameter		Total (%)
**TCGA**		***n* = 370**

Survival status	Alive	244 (65.9)
	Dead	126 (34.1)
Age	≤65 years	231 (62.4)
	>65 years	138 (37.3)
Gender	Male	249 (67.3)
	Female	121 (32.7)
Histological grade	G1	55 (14.9)
	G2	177 (47.8)
	G3	121 (32.7)
	G4	12 (3.2)
Stage	I	170 (45.9)
	II	86 (23.2)
	III	85 (23.0)
	IV	5 (1.4)
T	T1	180 (48.6)
	T2	94 (25.4)
	T3	80 (21.6)
	T4	13 (3.5)
N	N0	251 (67.8)
	N1	4 (1.1)
	NA	115 (31.1)
M	M0	265 (71.6)
	M1	4 (1.1)
	NA	101 (27.3)

**GSE14520 in GEO**		***n* = 221**

Survival status	Alive	136 (61.5)
	Dead	85 (38.5)
Gender	Male	191 (86.4)
	Female	30 (13.6)
Age	≤65 years	200 (90.5)
	>65 years	21 (9.5)
Main tumor size	>5 cm	80 (36.2)
	≤5 cm	140 (63.3)
MultiNodular	No	176 (79.6)
	Yes	45 (20.4)
TNM staging	I	93 (42.1)
	II	77 (34.8)
	III	49 (22.2)
BCLC staging	0	20 (9.0)
	A	148 (67.0)
	B	22 (10.0)
	C	22 (10.0)
CLIP staging score	0	97 (43.9)
	1	94 (42.5)
	2	35 (15.8)
	3	9 (4.1)
	4	3 (1.4)
	5	1 (0.5)

### Screening Metabolism-Related Differentially Expressed Genes

We downloaded the KEGG gene sets (c2.cp.kegg.v7.0.symbols.gmt) from the Molecular Signature Database (MSigDB) and extracted genes in amino acid metabolism pathways to find amino acid metabolic genes from the training set. Next, the intersecting amino acid metabolic genes in the validation set and the training set were selected, and their expression was corrected by the “sva” package (R software version 4.0.2) for further differential analysis. The “limma” package was employed to obtain differentially expressed genes (DEGs) between normal liver tissues and HCC from the training set. FDR < 0.05 and | logFC| ≥ 0.5 were the criteria used to define DEGs.

### Building and Validating the Amino Acid Metabolism-Related Prognostic Signature

To select genes significantly related to patient overall survival (OS) in the training set, a univariate Cox proportional hazard regression analysis was carried out. To ensure accurate results, patients with a survival time of less than 30 days were excluded. To prevent overfitting of the model, LASSO regression was carried out by “glmnet” R package. Genes with independent prognostic values were selected and the risk score formula was as follows:

$$\begin{array}{c} Riskscore=\left(CoefficientmRNA1\times expressionofmRNA1\right)\\ +\left(CoefficientmRNA2\times expressionofmRNA2\right)\\ +\cdots +\left(CoefficientmRNAn\times expressionmRNAn\right). \end{array}$$

According to the median risk scores, HCC patients were divided into high-risk and low-risk groups in both the training set and the validation set. Univariate and multivariate Cox proportional hazard regression analyses of risk scores and clinicopathological items were conducted to validate the performance of the prognostic signature. We generated Kaplan-Meier(K-M) curves using the “survival” and “survminer” R packages. To evaluate the predictive performance of the risk score for the 1-, 3-, and 5- year survival of HCC patients, we plotted a time-dependent receiver operating characteristic (ROC) curve with the “timeROC” and “survival” R packages.

### Establishing Predictive Nomograms

The results of the multivariate analysis were used to build nomograms for predicting 1-, 2-, 3-, and 5- year survival. The “rms” R package was employed to establish and visualize the results. The discrimination performance and predicting value of nomograms were assessed by Harrell’s C-index and calibration curve.

### Functional Enrichment Analysis

Gene set enrichment analyses (GSEA) were analyzed according to the Molecular Signatures Database (MSigDB, version 7.2) to reveal the molecular mechanism of the prognostic gene signature. The “c2 KEGG gene set,” and “c5 all GO gene sets” were chosen for analysis. GSEA software (version 4.1.0) was employed and the parameters were as follows: number of permutations = 1,000, min size = 15 and max size = 500. Pathways with NOM *p*-value < 0.05 and FDR *q*-value < 0.25 were defined as significantly enriched. The results were visualized by the “ggplot2” R package. The infiltration scores of 22 immune cells in the training set and validation set were analyzed with the CIBERSORTx web tool. The algorithm was run using the LM22 signature matrix at 1,000 permutations.

### Cell Culture and Treatment

The human hepatocyte LO2 cell line, human hepatoma HepG2 and Hep3B cell lines were obtained from the America Type Culture Collection (ATCC, Manassas, VA, United States). The cells were incubated at 37°C in a humid atmosphere containing 5% CO_2_. The cells were cultured in dulbecco’s modified eagle’s medium (DMEM) supplemented with 10% fetal bovine serum (FBS) (GIBCO-BRL, Thermo Fisher Scientific, Waltham, MA, United States). Cells were inoculated in 12-well plates and 6-well plates at densities of 1^∗^10^5^/well and 2^∗^10^5^/well, respectively.

### Quantitative Real-Time PCR

Total RNA from the cells were extracted by RNA isolater Total RNA Extraction Reagent (Vazyme, Nanjing, China) and were used to synthesis cDNA by HiScript II Q RT SuperMix for qPCR (Vazyme, Nanjing, China). To measure the abundance of mRNA, the cDNA template, primers (Supplementary Table 1) and AceQ qPCR SYBR Green Master Mix (Vazyme, Nanjing, China) were mixed and run in a Light-Cycler 480 Software (Roche Diagnostics GmbH, Mannheim, Germany). Glyceraldehyde-3-phosphate dehydrogenase (GAPDH) was used as an internal control. The 2^–ΔΔ*Ct*^ method was used to calculate mRNA expression.

### Western Blot Analyses

Total protein from the cells was extracted and concentrations were quantitated. Protein samples were mixed with 1 × SDS-polyacrylamide gel electrophoresis(SDS-PAGE) loading buffer and boiled for 10 min. Denatured proteins were separated by 10% polyacrylamide gels (EpiZyme, Shanghai, China). The separated proteins were transferred to polyvinylidene fluoride (PVDF) membranes (Millipore Corp., Billerica, MA, United States). The membranes were blocked with 8% non-fat milk prepared with TBST containing 0.1% Tween20 for 1 h, and incubated in the diluted specific antibodies (Supplementary Table 2) at 4°C overnight with gentle shaking, and the next day were incubated with the secondary antibody for 1 h. The immunoreactive bands were detected with enhanced chemiluminescence (ECL) kit (Vazyme, Nanjing, China) and quantified with ImageJ software (V1.8.0, National Institutes of Health).

### Immunohistochemistry Analysis

A total of three pairs of HCC and paired adjacent tissues were obtained from three patients of Union Hospital, Tongji Medical College, Huazhong University of Science and Technology. This clinical trial is registered in the Chinese Clinical Trial Registry (ChiCTR2100049106). The histologic grades of all HCC tissues were identified by the pathology department. Liver tissues were fixed with 4% paraformaldehyde, embedded in paraffin and made into 4 μm slices. Slices were dewaxed and incubated with diluted specific primary antibodies at 4°C overnight (Supplementary Table 3) and were subsequently incubated with biotinylated secondary antibody (Proteintech, Wuhan, China) at room temperature for 1 h. DAB chromogenic reagent was used to detect positive staining, and each section was counterstained with hematoxylin. An optical microscope (Olympus, BX-51, Tokyo, Japan) was used to take 40 × immunohistochemical images. For semi-quantitative immunostaining analysis, 5 random fields per slice were used to calculate average optical density, and ImageJ software (V1.8.0, National Institutes of Health) was used for analysis.

### Statistical Analysis

All statistical analyses were conducted by R software v4.0.2 and Graphpad Prism software v8.0.2. “Wilcox test” was used to compare gene expression and immune scores between different groups. Univariate Cox and LASSO regression analyses were adopted to identify the prognostic signature. The OS and recurrence-free survival (RFS) were analyzed by Kaplan-Meier analysis with a log-rank test. Gene expression at different stages was compared with one-way ANOVA. Immunohistochemical statistical analysis and gene and protein expression in different cells were analyzed by unpaired *t*-test. *P* < 0.05 was considered statistically significant.

## Results

### Building a Nine-Gene Amino Acid Metabolism-Related Risk Signature

**FIGURE 1:**
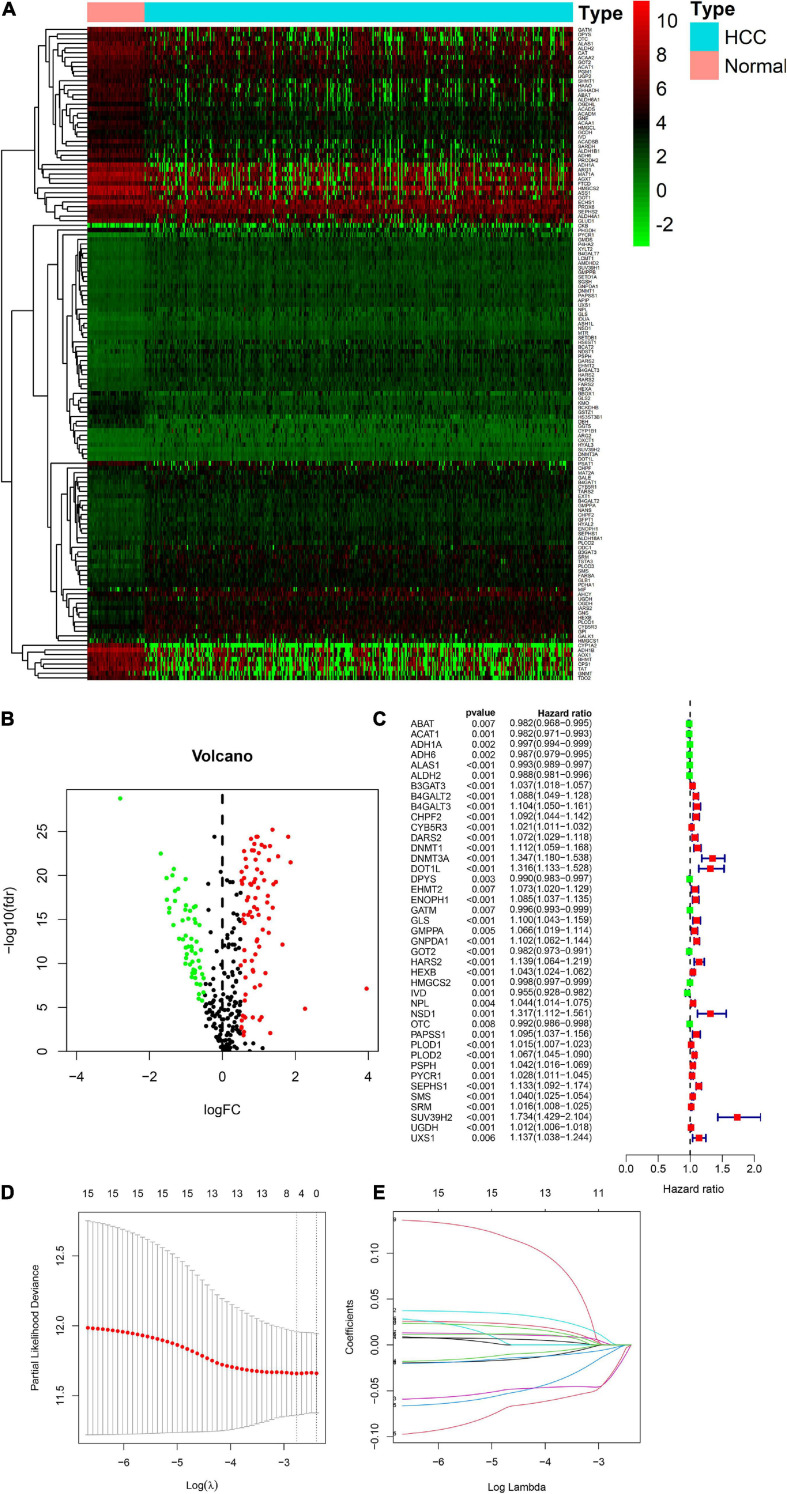
Visualization of differential amino acid metabolism-related genes. **(A)** The heatmap of differential amino acid metabolism-related genes. **(B)** The volcano plot of differential amino acid metabolism-related genes. **(C)** The forest plot of univariate Cox regression of OS related amino acid metabolism-related genes. **(D,E)** LASSO regression analysis.

There were 393 amino acid metabolic genes in the training set, and 327 amino acid metabolism-related genes were obtained after intersecting with the validation set. Further differential expression analysis showed that there were 140 differentially expressed genes (DEGs) in the training set (82 upregulated and 58 downregulated, Supplementary Table 4). The heatmap and volcano plot of 140 DEGs are drawn in Figures 1A,B. Next, we adopted the univariate Cox proportional hazard regression analysis to identify mRNAs related to OS. The 41 genes with prognostic value (*p* < 0.01) in univariate Cox regression were further analyzed by LASSO regression (Supplementary Table 5 and Figures 1C–E). The model was constructed by using the “glmnet” and “survival” R packages. Finally, 9 amino acid metabolism-related genes were chosen to establish a prognostic model, including B3GAT3, B4GALT2, CYB5R3, GNPDA1, GOT2, HEXB, HMGCS2, PLOD2, and SEPHS1. Detailed information on nine genes is listed in Table 2. The risk score formula was as follows: Risk score = (0.0068^∗^expression_*B*__3G__*AT*__3_) + (0.0123^∗^ expression_*B*__4G__*ALT*__2_) + (0.0001^∗^expression_*CYB*__5R__3_) + (0.0072^∗^ expression_*GNPDA*__1_)—(0.0006^∗^ expression_*GOT*__2_) + (0.0071^∗^ expression_*HEXB*_)—(2.5286e-05^∗^ expression_*HMGCS*__2_) + (0.0181^∗^ expression_*PLOD*__2_) + (0.0601^∗^ expression_*SEPHS*__1_).

**TABLE 2:** Detail information of nine genes in the risk signature.

Gene symbol	Full name	Function of the encoded protein
B3GAT3	Beta-1, 3-glucuronyltransferase 3	B3GAT3 is a glycosyltransferase that plays an important role in proteoglycan (PG) biosynthesis
B4GALT2	Beta-1, 4-galactosyltransferase 2	B4GALT2 is a beta-1, 4-galactosyltransferase (beta4GalT) and synthesizes N-acetyllactosamine in glycolipids and glycoproteins
CYB5R3	Cytochrome b5 reductase 3	CYB5R3 encodes cytochrome b5 reductase, functions in desaturation and elongation of fatty acids, in cholesterol biosynthesis, and in drug metabolism
GNPDA1	Glucosamine-6-phosphate deaminase 1	GNPDA1 links the hexosamine system with the glycolytic pathway and promotes the catabolism of hexosamines derived from glycoproteins, glycolipids, and sialic acids into phosphate sugars to provide energy sources
GOT2	Glutamic-oxaloacetic transaminase 2	GOT plays a role in amino acid metabolism and the urea and tricarboxylic acid cycles
HEXB	Hexosaminidase subunit beta	HEXB catalyzes the degradation of the ganglioside GM2, and other molecules containing terminal N-acetyl hexosamines
HMGCS2	3-hydroxy-3-methylglutaryl-CoA synthase 2	HMGCS2 is a mitochondrial enzyme that catalyzes the first reaction of ketogenesis, a metabolic pathway that provides lipid-derived energy for various organs during times of carbohydrate deprivation
PLOD2	Procollagen-lysine	PLOD2 catalyzes the hydroxylation of lysyl residues in collagen-like peptides
SEPHS1	Selenophosphate synthetase 1	SEPHS1 is an enzyme that synthesizes selenophosphate from selenide and ATP

### Validating the Amino Acid Metabolism-Related Risk Signature

**FIGURE 2:**
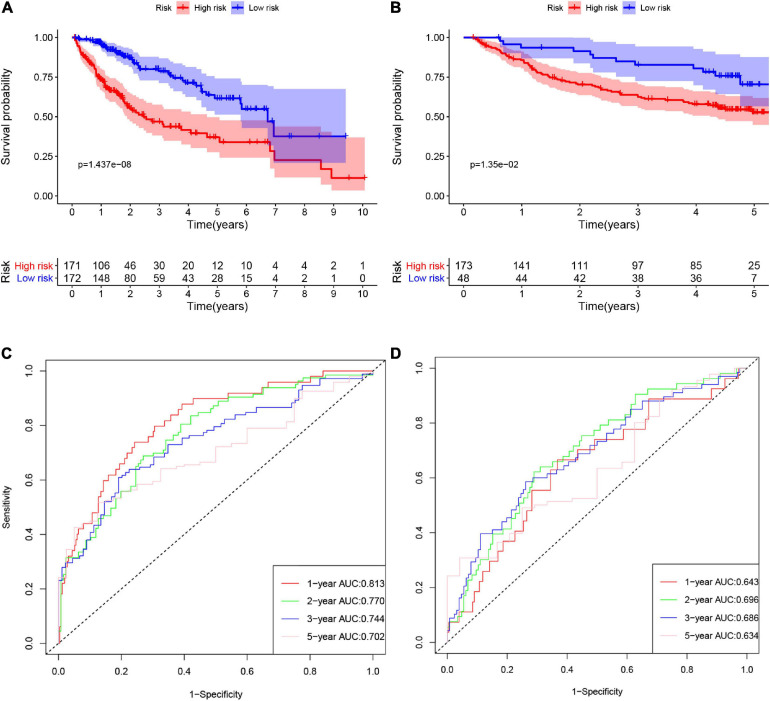
The K-M curves of high-risk and low-risk groups in the training set **(A)** and validation set **(B)**. The ROC curves of risk signature’s predicting performance in 1-, 2-, 3-, and 5-year survival for HCC patients in the training set **(C)** and validation set **(D)**.

**FIGURE 3:**
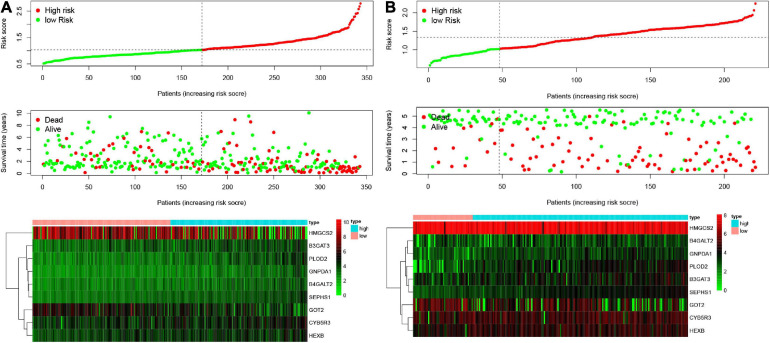
Characteristics of risk scores and heatmaps of the amino acid metabolism-related gene signature. **(A)** The risk score, survival time, and status of patients in the training set. **(B)** The risk score, survival time, and status of patients in the validation set.

**FIGURE 4:**
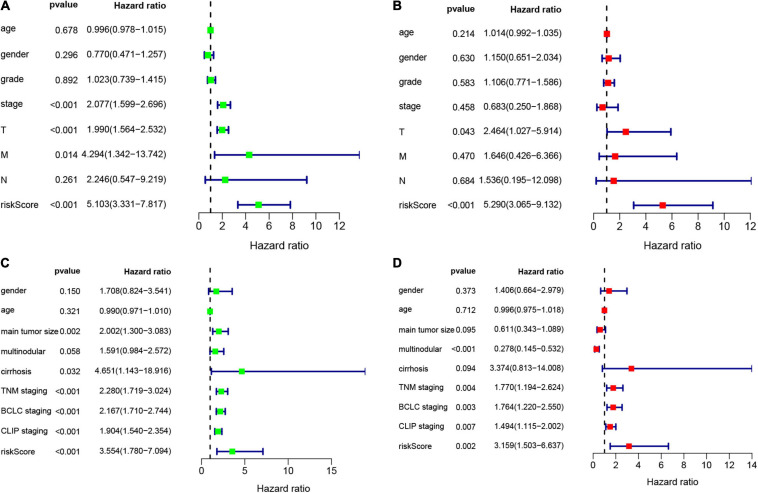
Univariate and multivariate Cox analysis to evaluate independent prognostic value of the risk signature. **(A)** Univariate Cox analysis in the training set. **(B)** Multivariate Cox analysis in the training set. **(C)** Univariate Cox analysis in the validation set. **(D)** Multivariate Cox analysis in the validation set.

According to the formula, we calculated the risk scores of patients in the training set and validation set. Then we separated patients into high-risk and low-risk groups according to the median risk score. The K-M curves showed worse OS in the high-risk group than in the low-risk group in both the training set and validation set (*p* = 1.437e−08, and *p* = 1.35e−02, respectively) (Figures 2A,B). In Figure 3, we plotted heatmaps of gene expression and displayed the impact of risk scores on risk ranking, survival time and survival status. The results verified that the signature had significant prognostic value for HCC patients. The independent prognostic analysis of the univariate and multivariate Cox proportional hazard regression also verified the effective prognostic value (Figure 4). Furthermore, ROC curves were drawn to assess the efficiency of risk scores in predicting 1-, 2-, 3-, and 5- year survival. In the training set, the AUCs for 1-, 2-, 3-, and 5- year survival was 0.813 (*P* < 0.001, 95%CI:0.748–0.878), 0.770 (*P* < 0.001, 95%CI:0.705–0.835), 0.744 (*P* < 0.001, 95%CI:0.6690.820) and 0.702 (*P* < 0.001, 95%CI:0.611–0.793), respectively. In the validation set, the AUCs for 1-, 2-, 3-, and 5- year survival was 0.643 (*P* = 0.013, 95%CI:0.530–0.757), 0.696 (*P* < 0.001, 95%CI:0.615–0.777), 0.686 (*P* < 0.001, 95%CI:0.606–0.765) and 0.634 (*P* = 0.027, 95%CI:0.515−0.752), respectively (Figures 2C,D).

### Validating the Nine Genes in External Databases

**FIGURE 5:**
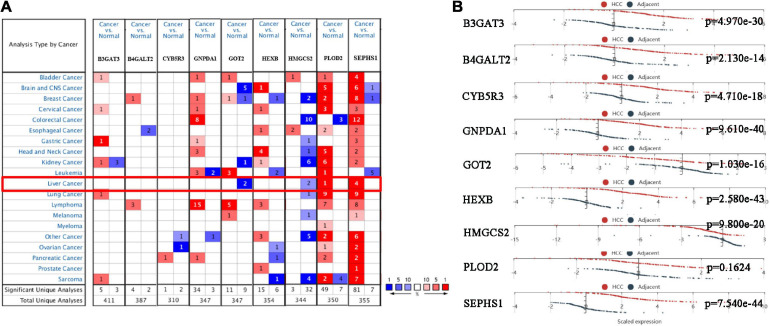
The mRNA expression of the prognostic genes in HCC patients. **(A)** mRNA expression of the prognostic genes in 20 cancers from the Oncomine database. **(B)** mRNA expression of the prognostic genes between HCC and normal tissues in the HCCDB database.

We analyzed the expression of nine genes in twenty types of cancers in the Oncomine database. The thresholds were as follows: *p*-value = 1E-4, fold change = 2, gene rank = Top 10%, and data type = mRNA. Nine genes were altered in different cancers (Figure 5A). GOT2 and HMGCS2 were downregulated in HCC, while PLOD2 and SEPHS1 were upregulated in HCC. The HCCDB database curated 15 public HCC expression datasets, among which the HCCDB18 dataset contained RNA-seq of 212 tumor and 177 adjacent normal tissues obtained from the ICGC-LIR-JP cohort. As shown in Figure 5B, B3GAT3, B4GALT2, CYB5R3, GNPDA1, HEXB, and SEPHS1 were significantly upregulated in HCC samples, while GOT2 and HMGCS2 were downregulated.

**FIGURE 6:**
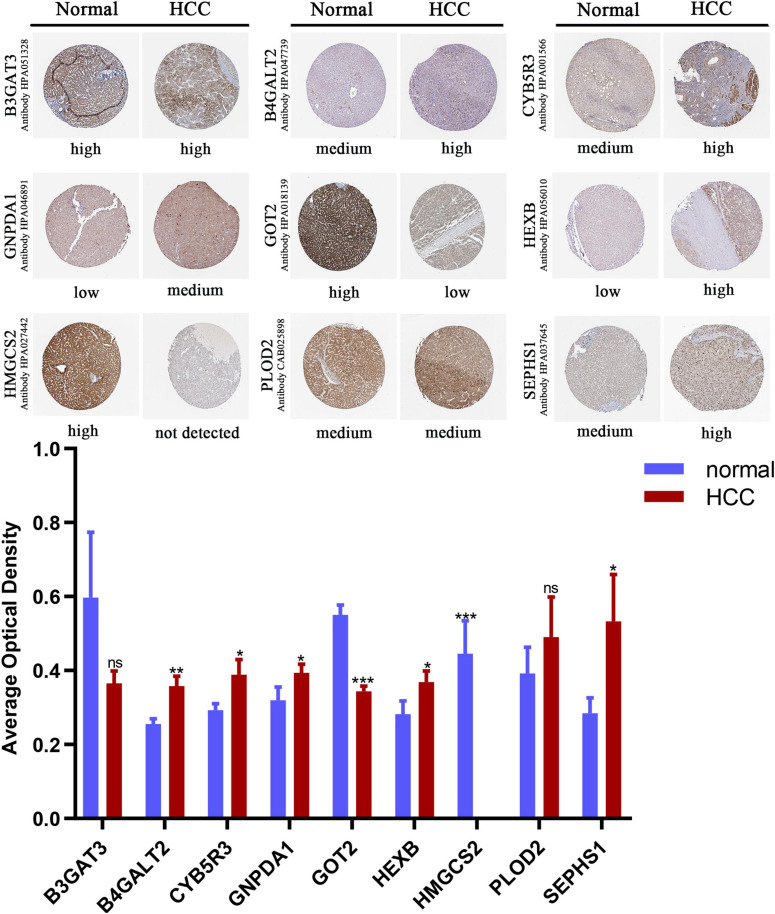
Immunohistochemistry staining of the prognostic genes in HCC and normal liver tissues (the HPA database). Data are shown as mean ± SD, *p < 0.05, **p < 0.01, ***p < 0.001 vs normal liver tissues.

We compared the protein expression encoded by the nine genes between HCC and normal liver tissues in the Human Protein Atlas (HPA) database. Consistent with the mRNA expression levels, GOT2 and HMGCS2 decreased in HCC tissues, and B4GALT2, CYB5R3, GNPDA1, HEXB, and SEPHS1 increased in HCC tissues. B3GAT3 and PLOD2 had no differential expression (Figure 6).

**FIGURE 7:**
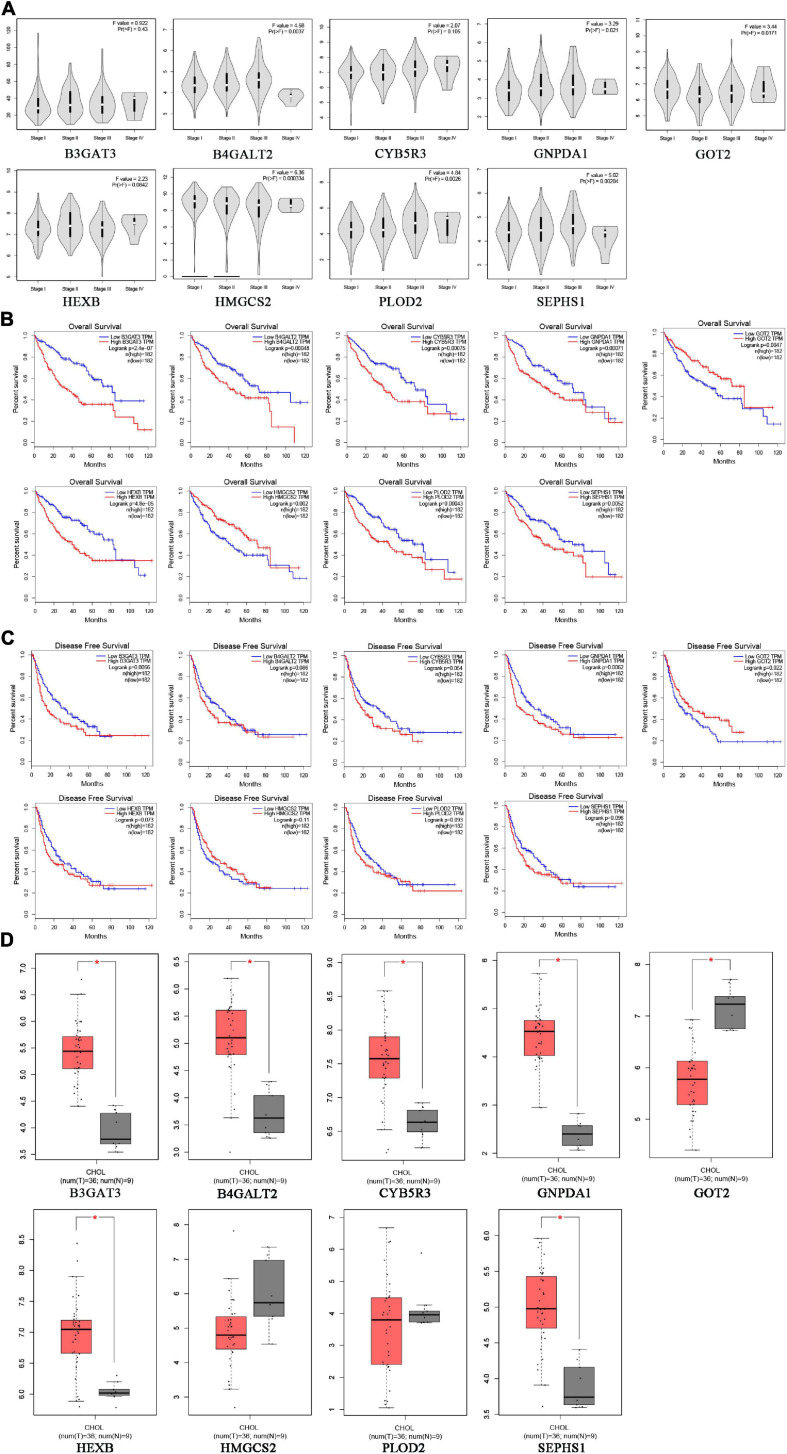
The expression of prognostic genes in HCC and CHOL analyzed based on the GEPIA database. **(A)** The mRNA expression from stage I to stage IV in HCC patients. **(B)** The prognostic value of OS in HCC patients. **(C)** The prognostic value of RFS in HCC patients. **(D)** The mRNA expression in 9 normal liver tissues and 36 CHOL tissues.

To verify the clinical performance of these nine genes, we discussed the relationship between mRNA levels and pathological stages according to the Gene Expression Profiling Interactive Analysis (GEPIA) database. GOT2 and HMGCS2 were gradually downregulated from stage I to stage IV, while B4GALT2, GNPDA1, PLOD2 and SEPHS1 were gradually upregulated from stage I to stage III (Figure 7A). We also explored the influence of each gene on OS and recurrence-free survival (RFS) of HCC patients. High expression of GOT2, HMGCS2 and low expression of B3GAT3, B4GALT2, CYB5R3, GNPDA1, HEXB, PLOD2, SEPHS1 correlated with favorable OS (Figure 7B). In addition, low expression of B3GAT3 and GNPDA1 and high expression of GOT2 correlated with favorable RFS (Figure 7C).

Cholangiocarcinoma (CHOL) is the second most common subtype of liver cancer. To identify whether the prognostic signature is specific for HCC, we compared the expression of nine genes between CHOL and normal liver tissues based on the GEPIA database. B3GAT3, B4GALT2, CYB5R3, GNPDA1, HEXB, SEPHS1 were upregulated and GOT2 was downregulated in CHOL, which was consistent with expression in HCC (Figure 7D). However, PLOD2 and HMGCS2 had no difference between CHOL and normal liver tissues. These results may indicate that the risk signature is specific for HCC rather than other subtypes of liver cancer.

### Creating Predictive Nomograms

**FIGURE 8:**
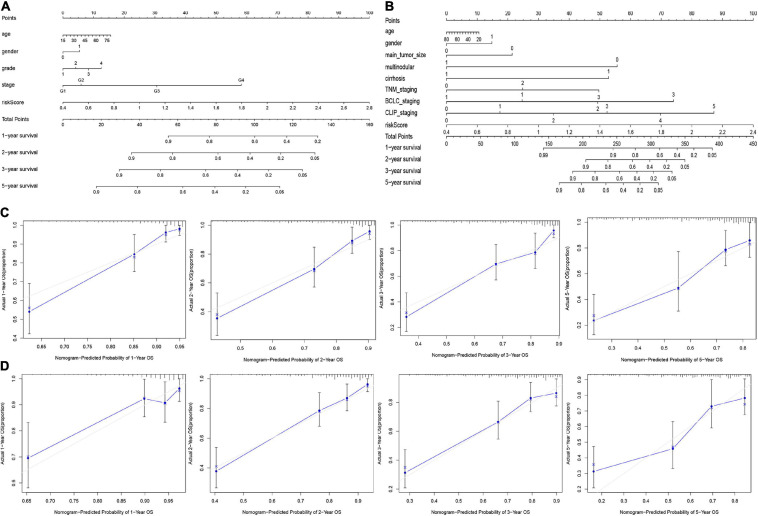
Nomograms for predicting the OS of 1-, 2-, 3-, and 5-years in the training set **(A)** and validation set **(B)**. Calibration plot of nomograms in the training set **(C)** and the validation set **(D)**.

Based on the final regression analysis, a nomogram was created that incorporated a 9-gene risk signature and clinicopathological parameters. In the training set, age, gender, grade, stage and risk score were chosen in the final model (Figure 8A). In the validation set, age, gender, main tumor size, multinodular status, TNM staging, BCLC staging, CLIP staging and risk score were chosen in the final model (Figure 8B). Both of the nomograms displayed good discrimination performance. The C-index were 0.786 (95%CI: 0.734–0.838) and 0.722 (95%CI: 0.665–0.779) in the training set and validation set, respectively. Besides, calibration plots showed that nomograms of both the training set and validation set had good agreements between the prediction and actual clinical survival outcomes (Figures 8C,D). A total score could be calculated to measure the 1-, 2-, 3-, and 5-year survival rates of HCC patients.

### Functional Enrichment Analysis

**FIGURE 9:**
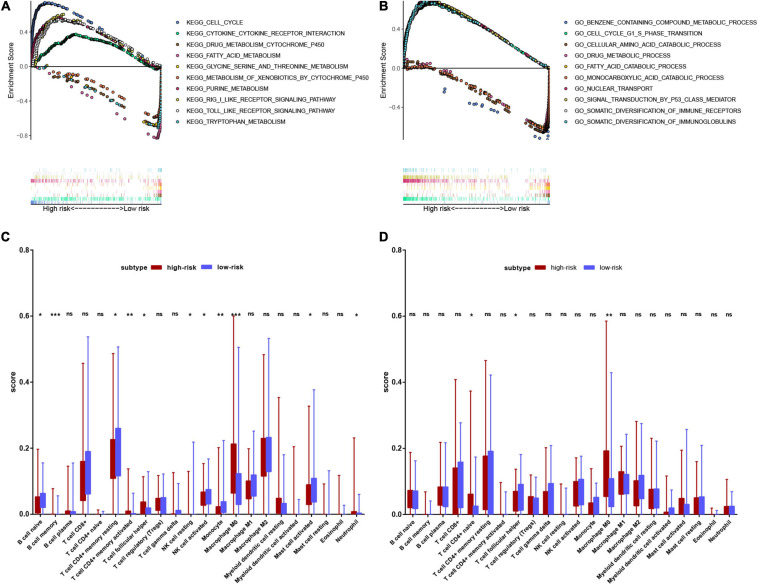
Functional enrichment analysis of genes between high-risk and low-risk groups. **(A)** Result of GSEA analysis in the training set. **(B)** Result of GSEA analysis in the validation set. **(C)** Result of the infiltrating score of 22 immune cells in the training set. **(D)** Result of the infiltrating score of 22 immune cells in the validation set.

We performed GSEA to clarify the enrichment pathways of the low-risk and high-risk groups in the training set. In the high-risk group, KEGG pathways were mainly enriched in the cell cycle, nucleotide metabolism, and immune-related pathways, including RIG-I like receptor signaling pathway, Toll-like receptor signaling pathway, and cytokine-cytokine receptor interaction. In the low-risk group, KEGG pathways mainly enriched in drug metabolism-cytochrome P450, amino acid metabolism and fatty acid metabolism (Figure 9A). In the high-risk group, biological processes were mostly enriched in the cell cycle, nuclear transport, P53 signaling, and diversification of immune molecules, and in the low-risk group, biological processes were mainly enriched in amino acid and fatty acid catabolic processes, toxin and drug metabolic processes (Figure 9B).

We subsequently explored the connection between the risk score and immune status. According to the CIBERSORTx database, we compared the infiltration scores of 22 immune cells in the low-risk group and the high-risk group. In the training set, the high-risk group had significantly higher infiltration of memory B cells, activated memory CD4^+^ T cells, T follicular helper (Tfh) cells, M0 macrophages, and neutrophils, while the low-risk group had significantly higher infiltration of naive B cells, resting memory CD4^+^ T cells, resting natural killer (NK) cells, activated NK cells, monocytes and activated mast cells (Figure 9C). In the validation set, the high-risk group had significantly higher scores of naive CD4^+^ T cells and M0 macrophages, and a lower score of Tfh cells (Figure 9D).

### Validating the Nine Genes in Cells and Human Liver Tissues

**FIGURE 10:**
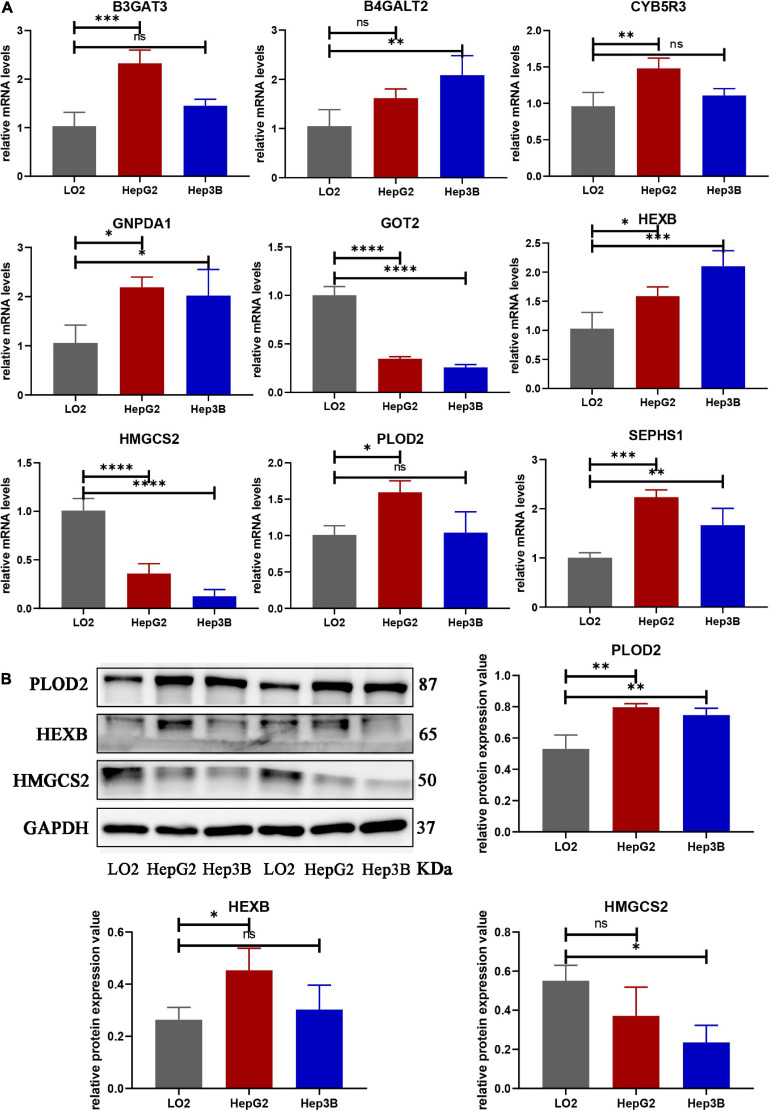
The mRNA and protein expression of prognostic genes in LO2 cells, HepG2 cells and Hep3B cells. **(A)** The mRNA expression of nine genes analyzed by qRT-PCR. **(B)** The total protein expression of PLOD2, HEXB and HMGCS2 analyzed by western blot. Data are shown as mean ± SD, ^∗^*p* < 0.05, ^∗∗^*p* < 0.01, ^∗∗∗^*p* < 0.001, ^****^p < 0.0001 vs. LO2 cells.

We compared the mRNA levels of nine genes between the LO2 cell line and HCC cell lines (HepG2 and Hep3B) by qRT-PCR analysis. As shown in Figure 10A, expression of B3GAT3, CYB5R3, and PLOD2 were significantly higher in HepG2 cells than in LO2 cells, expression of B4GALT2 was significantly higher in Hep3B cells than in LO2 cells, and expression of GNPDA1, HEXB, SEPHS1 were significantly higher in both HepG2 and Hep3B cells compared with LO2 cells.

**FIGURE 11:**
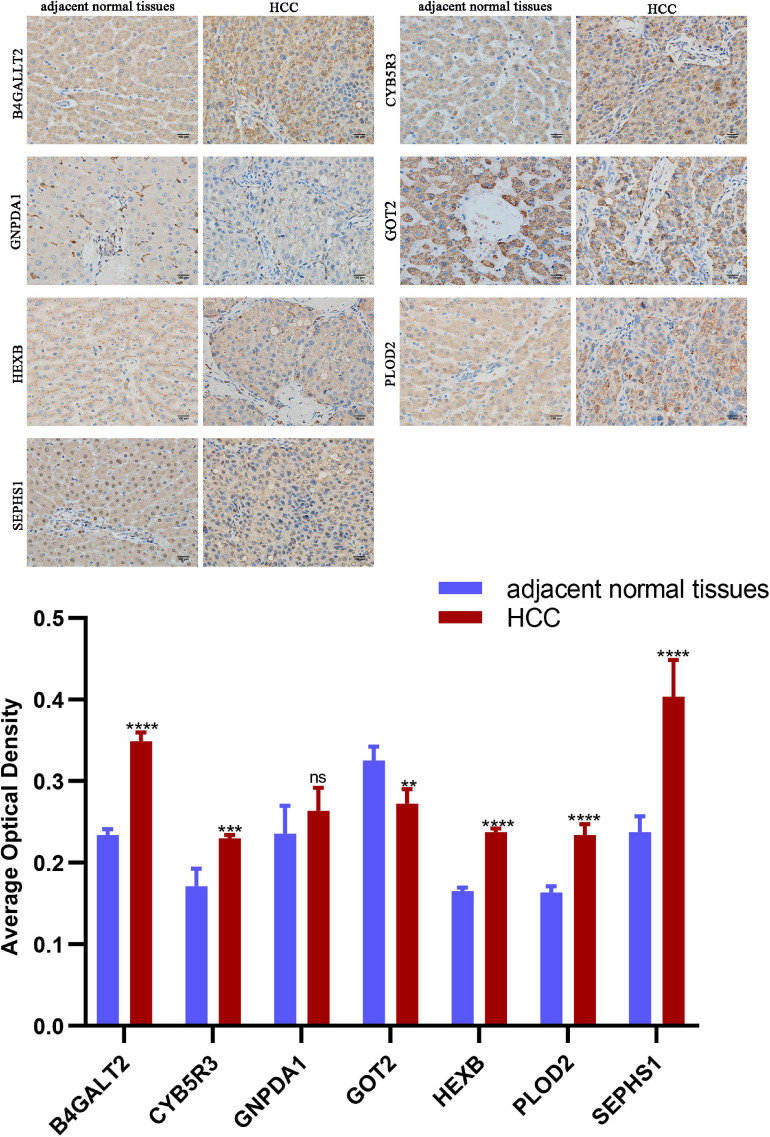
Representative images of immunohistochemistry staining of prognostic genes in HCC and adjacent normal tissues. Data are shown as mean ± SD, **p < 0.01, ***p < 0.001, ****p < 0.0001 vs adjacent normal tissues.

Protein expression analyzed by western blot showed that PLOD2 was significantly upregulated in HepG2 and Hep3B cells compared with LO2 cells, HEXB was significantly upregulated in HepG2 cells compared with LO2 cells, and HMGCS2 was significantly downregulated in Hep3B cells compared with LO2 cells (Figure 10B). Besides, immunohistochemistry analysis revealed that B4GALT2, CYB5R3, HEXB, PLOD2, and SEPHS1 were significantly upregulated while GOT2 was significantly downregulated in HCC tissues compared with adjacent normal tissues (Figure 11).

## Discussion

Metabolism has been revealed to be closely correlated with epigenetics in cancer in recent years (Vander and DeBerardinis, 2017; Thakur and Chen, 2019). Aberrant metabolism promotes tumor proliferation and metastasis. Many metabolism-related genes have proven to be effective prognostic biomarkers. HCC has active metabolic reprogramming and amino acid metabolism is an important metabolic variation. Several studies have explored the energy metabolism-related risk signatures of HCC through bioinformatic methods (Zou et al., 2019; Liu et al., 2020; Tang et al., 2020). However, there is still a lack of bioinformatic research on amino acid metabolism-related genes in HCC. In this study, for the first time, we analyzed the characteristics of amino acid metabolism-related genes in HCC and built a risk signature correlated with OS. First, we identified 140 amino acid metabolism-related DEGs between HCC and normal liver tissues based on RNA-seq data. Next, we built an effective prognostic signature based on univariate Cox and LASSO regression. Nine genes are contained in this signature (B3GAT3, B4GALT2, CYB5R3, GNPDA1, GOT2, HEXB, HMGCS2, PLOD2, and SEPHS1).

Patients can be divided into high-risk and low-risk groups according to risk scores. K-M curves showed that low-risk patients had a longer survival time than high-risk patients. Furthermore, the ROC curve confirmed that the risk signature can effectively predict the 1-, 2-, 3-, and 5- year survival rates of HCC patients. Univariate and multivariate Cox analyses confirmed the independent prognostic value of the risk signature. Several studies in recent years have identified metabolism-related risk signatures for effectively predicting OS and have uncovered the importance of metabolism-related genes in the process of HCC development (Hu et al., 2020; Wu et al., 2021). Zhu’s study built a lipid metabolism-related prognostic signature and revealed that lipid metabolism-related genes are closely correlated with clinical characteristics, immune cells, and multiple biological functions of HCC (Zhu et al., 2021). However, this study did not build a predictive nomogram. Our study not only built the risk signature but also built nomograms based on the training set and validation set. Wu’s study (Wu et al., 2021) built a six-gene metabolism risk signature, which mainly focuses on nucleotide metabolism and lipid metabolism. However, our study mainly focuses on amino acid metabolism. Liu’s study built an amino acid metabolism-related prognostic signature of glioma and verified that risk scores closely correlated to different aspects of the malignancy of glioma (Liu et al., 2019). Consistently, our study verified the importance of energy metabolism disorders in HCC. We also emphasized that the amino acid metabolism-related genes played a vital role in the process of HCC development. Our study built a 9-gene signature that could effectively predict the OS of HCC patients in both the training set and validation set, and more importantly, it is a risk factor that is independent of clinicopathological factors.

Beta-1, 3-glucuronyltransferase 3 (B3GAT3) promotes the proliferation, metastasis and epithelial mesenchymal transition (EMT) process of the human HepG2 liver cancer cell line (Zhang Y. L. et al., 2019). Selenophosphate synthetase 1 (SEPHS1) promotes the expression of SMADs in liver cancer cells and stimulates the migration and invasion of tumors induced by TGF-β, which is negatively correlated with the OS and RFS of HCC patients (Yang et al., 2021). Glucosamine-6-phosphate deaminase 1 (GNPDA1) and procollagen-lysine (PLOD2) are upregulated in liver cancer and promote tumor proliferation and migration, which are correlated with poor prognosis (Du et al., 2017; Xia et al., 2021). Mutation of Beta-1, 4-galactosyltransferase 2 (B4GALT2) leads to abnormal glycosylation of proteins, promoting the development of colon cancer (Venkitachalam et al., 2016). Downregulation of glutamic-oxaloacetic transaminase 2 (GOT2) activates oxidative stress in human pancreatic ductal adenocarcinoma (PDAC) cells and inhibits the proliferation of pancreatic cancer (Yang et al., 2018). Downregulation of 3-hydroxy-3-methylglutaryl-CoA synthase 2 (HMGCS2) reduces ketone production, enhances the c-Myc/cyclinD1 and EMT signaling pathways, and inhibits caspase-dependent apoptosis pathways, which promotes tumor proliferation, migration and xenograft tumorigenesis in various cancers (Tang et al., 2017). The function of membrane-bound cytochrome b5 reductase 3 (CYB5R3) and hexosaminidase subunit beta (HEXB) on cancers is still unclear.

Combining several databases, our study found that the mRNA levels of B3GAT3, B4GALT2, CYB5R3, GNPDA1, HEXB, PLOD2, and SEPHS1 were increased in HCC tissues while mRNA levels of GOT2 and HMGCS2 were decreased in HCC tissues. In addition, the protein expression was consistent with the mRNA expression. Moreover, the expression of nine genes correlated with the prognosis of HCC patients. Interestingly, we noticed that PLOD2 and HMGCS2 had no difference between CHOL and normal liver tissues. These results may indicate that the risk signature is specific for HCC rather than other subtypes of liver cancer. We also verified the mRNA and protein expression of nine genes in HCC patient samples and cell lines, which showed consistent results with the public database mining results. In brief, our results verified the importance of B3GAT3, SEPHS1, PLOD2, and GNPDA1 in HCC development again. However, previous studies have lacked an exploration of B4GALT2, GOT2, and HMGCS2 changes in HCC. Our study disclosed the prognostic value of B4GALT2, GOT2, and HMGCS2 in HCC. CYB5R3 functions in drug metabolism, cholesterol biosynthesis, desaturation of fatty acids, and mitochondrial electron transport chain (ETC) activity (Fan et al., 2020). HEXB participates in catalyzing the degradation of the ganglioside GM2 (Kuil et al., 2019). No influence of two genes (CYB5R3 and HEXB) in cancers has been reported, but our study found that upregulation of CYB5R3 and HEXB was significantly linked to the poor prognosis of HCC patients. Next, we developed nomograms for predicting the 1-, 2-, 3-, and 5-year OS of HCC patients according to risk scores and clinicopathological characteristics.

We further performed pathway enrichment analysis. GSEA revealed that cell cycle regulation and synthesis of biological macromolecules were active in high-risk patients, which discloses the importance of amino acid metabolism-related genes in the energy metabolism process for tumor proliferation. Amino acid and fatty acid catabolic processes and toxin and drug metabolic processes were inhibited in high-risk patients, which indicates that amino acid metabolism-related genes may participate in tumorigenesis. We also noticed that immune response-related pathways were enriched. In our study, infiltration of memory B cells, activated memory CD4^+^ T cells, T follicular helper (Tfh) cells, and naive CD4^+^ T cells was upregulated while infiltration of naive B cells was downregulated in high-risk patients. It was reported that there was more B cells infiltration in HCC patients than in patients with cirrhosis and healthy people (Zhang et al., 2020). Upregulation of plasma cells and lower expression of naive B cells correlated with poorer prognosis (Zhang Z. et al., 2019). In addition, recent studies found that lower expression of CD8^+^ T cells can lead to immune dysfunction in HCC patients. Higher expression of Treg cells can interfere with cell cycle checkpoints and inhibit effector T cells, and promote the progression of HCC, which are factors related to poor prognosis of HCC. Consistently, our results revealed that changes in amino acid metabolism-related genes influence the ratio of different B and T cell subtypes, leading to an influence on prognosis. Our findings also show that high-risk patients have lower NK cell infiltration. It was reported that increased catabolism of Trp and Arg can induce apoptosis of NK cells, leading to tumor immune escape (Grohmann and Bronte, 2010). Therefore, abnormal expression of amino acid metabolism-related genes may promote immune escape by influencing NK cells in HCC proliferation. In addition, the proportion of neutrophils and M0 macrophages increased in high-risk patients in this study. Neutrophils influence tumor progression by releasing cytokines and chemokines with tumorigenic or antitumor functions (Haider et al., 2019). Zhou’s study also found that neutrophils can recruit macrophages and Treg cells to promote HCC proliferation and drug resistance (Zhou et al., 2016). It was found that M0 and M1 macrophages were significantly correlated with RFS in HBV-HCC and HCV-HCC (Hsiao et al., 2019). Combined with our results, abnormalities in amino acid metabolism-related genes may affect neutrophil function and the interaction between neutrophils and macrophages to promote HCC proliferation.

## Conclusion

In conclusion, our study screens out amino acid metabolism-related genes which serve as potential prognostic biomarkers and builds a novel risk signature that is independently related to the overall survival of HCC. The findings provide an effective prediction of HCC prognosis and personalized therapy for liver cancer patients. The mechanisms related to amino acid metabolism-related genes and immune regulation during HCC development need further exploration.

## Data Availability Statement

The raw data supporting the conclusions of this article will be made available by the authors, without undue reservation.

## Ethics Statement

The studies involving human participants were reviewed and approved by the Medical Ethics Committee of Tongji Medical College of Huazhong University of Science and Technology. The patients/participants provided their written informed consent to participate in this study. Written informed consent was obtained from the individual(s) for the publication of any potentially identifiable images or data included in this article.

## Author Contributions

KX and JZ conceived and designed the study. YZ and SW performed the data analysis. YZ, JZ, and QJ wrote the manuscript. All authors read and approved the manuscript.

## Conflict of Interest

The authors declare that the research was conducted in the absence of any commercial or financial relationships that could be construed as a potential conflict of interest.

## Publisher’s Note

All claims expressed in this article are solely those of the authors and do not necessarily represent those of their affiliated organizations, or those of the publisher, the editors and the reviewers. Any product that may be evaluated in this article, or claim that may be made by its manufacturer, is not guaranteed or endorsed by the publisher.

## Footnotes

1
https://portal.gdc.cancer.gov/
