# Supplementary material for: Identification and Validation of a Nine-Gene Amino Acid Metabolism-Related Risk Signature in HCC
Source: Front Cell Dev Biol. 2021 Sep 7;9:731790. doi: 10.3389/fcell.2021.731790 (PMC8452960; doi:10.3389/fcell.2021.731790)
Supplement: Supplementary file 1 [file Data_Sheet_1.docx]

**Table.S1** Primers of quantitative real-time PCR

| Genes | Forward (5′->3′) | Reverse (5′->3′) |
| --- | --- | --- |
| B3GAT3 | AAGGAGTCGTCTACTTTGCTGA | GGGCATTGGGCTTATCTAACAG |
| B4GALT2 | CGCTACTGGCTCCACTATCTA | CCCGGTTGAAGGTGTCCTC |
| CYB5R3 | TCTACCTCTCGGCTCGAATTG | CCTTGTCATCATCGCTGGAGAT |
| GNPDA1 | CCTGGAGCACTATTCTCAGGC | CCTGGGTTAAACTGGATGATGC |
| GAPDH | ACAACTTTGGTATCGTGGAAGG | GCCATCACGCCACAGTTTC |
| GOT2 | AAGAGTGGCCGGTTTGTCAC | AGAAAGACATCTCGGCTGAACT |
| HEXB | GTCAGAGTGTGATGCTTTCCC | TAAACCTCGTAATGCTCCCCA |
| HMGCS2 | CAGTCCAAGAGGACATCAACTC | CAGTGCCTACTTCCAGCCTG |
| PLOD2 | GACAGCGTTCTCTTCGTCCTCA | CTCCAGCCTTTTCGTGGTGACT |
| SEPHS1 | GTAACAGGCGGCCAAACAGTA | GCACTGCATTGTCTGGCAT |

**Table.S2** Primary antibodies of western blot

| Primary antibody | Catalog number | Company |
| --- | --- | --- |
| GAPDH (1:5000) | ANT012 | Antgene, Wuhan, China |
| HMGCS2 (1:1000) | A19232 | Abclonal, Wuhan, China |
| HEXB (1:1000) | 16229-1-AP | Proteintech, Wuhan, China |
| PLOD2 (1:2000) | 66342-1-Ig | Proteintech, Wuhan, China |

**Table.S3** Primary antibodies of immunohistochemistry analysis

| Primary antibody | Catalog number | Company |
| --- | --- | --- |
| B4GALT2 (1:100) | A17573 | Abclonal, Wuhan, China |
| CYB5R3 (1:100) | 10894-1-AP | Proteintech, Wuhan, China |
| GNPDA1 (1:50) | 12312-1-AP | Proteintech, Wuhan, China |
| GOT2 (1:100) | 14800-1-AP | Proteintech, Wuhan, China |
| HEXB (1:50) | 16229-1-AP | Proteintech, Wuhan, China |
| PLOD2(1:200) | 66342-1-Ig | Proteintech, Wuhan, China |
| SEPHS1(1:100) | A6454 | Abclonal, Wuhan, China |

**Table.S4** Amino acid metabolism-related DEGs in the training set

| Gene name | _Normal_Mean | _HCC_Mean | logFC | p value | fdr |
| --- | --- | --- | --- | --- | --- |
| ABAT | 35.9263 | 16.8636 | -1.0911 | 3.54E-16 | 1.30E-15 |
| ACAA1 | 33.1314 | 16.5837 | -0.9984 | 4.88E-18 | 2.51E-17 |
| ACAA2 | 65.0000 | 28.0885 | -1.2105 | 5.18E-20 | 3.30E-19 |
| ACADM | 22.7523 | 14.4841 | -0.6515 | 2.07E-11 | 4.93E-11 |
| ACADS | 45.8581 | 17.7534 | -1.3691 | 9.34E-22 | 9.78E-21 |
| ACADSB | 46.4331 | 18.8767 | -1.2985 | 1.45E-18 | 7.70E-18 |
| ACAT1 | 52.5800 | 27.5388 | -0.9331 | 2.12E-16 | 8.18E-16 |
| ADH1A | 237.0610 | 85.9610 | -1.4635 | 3.72E-17 | 1.60E-16 |
| ADH1B | 321.0497 | 117.1296 | -1.4547 | 1.07E-17 | 5.07E-17 |
| ADH6 | 56.2081 | 26.5825 | -1.0803 | 6.09E-14 | 1.84E-13 |
| AGXT | 224.2516 | 129.8217 | -0.7886 | 3.90E-10 | 8.30E-10 |
| AHCY | 21.2812 | 39.3917 | 0.8883 | 2.94E-08 | 5.29E-08 |
| ALAS1 | 93.9249 | 52.5160 | -0.8388 | 1.79E-09 | 3.52E-09 |
| ALDH18A1 | 5.0581 | 10.8984 | 1.1074 | 4.28E-15 | 1.44E-14 |
| ALDH1B1 | 40.9604 | 22.1102 | -0.8895 | 5.74E-11 | 1.31E-10 |
| ALDH2 | 97.0823 | 38.6043 | -1.3304 | 1.46E-22 | 1.86E-21 |
| ALDH4A1 | 58.5348 | 38.4483 | -0.6064 | 1.34E-08 | 2.48E-08 |
| ALDH6A1 | 42.3724 | 17.2271 | -1.2984 | 9.30E-20 | 5.79E-19 |
| AMDHD2 | 2.7273 | 4.6375 | 0.7659 | 1.28E-20 | 9.05E-20 |
| AOX1 | 131.8676 | 66.1308 | -0.9957 | 1.02E-11 | 2.46E-11 |
| APIP | 3.4915 | 5.0300 | 0.5267 | 3.30E-22 | 3.86E-21 |
| ARG1 | 136.7334 | 89.6695 | -0.6087 | 1.46E-08 | 2.68E-08 |
| ARG2 | 2.1028 | 3.1720 | 0.5931 | 7.62E-05 | 0.0001 |
| ASH1L | 2.4753 | 3.7554 | 0.6014 | 1.22E-12 | 3.11E-12 |
| ASS1 | 353.7702 | 123.2981 | -1.5207 | 1.04E-18 | 5.74E-18 |
| B3GAT3 | 4.6691 | 12.9133 | 1.4676 | 6.76E-21 | 5.50E-20 |
| B4GALT2 | 4.1988 | 8.1234 | 0.9521 | 1.47E-17 | 6.83E-17 |
| B4GALT3 | 3.5713 | 7.9738 | 1.1588 | 2.07E-25 | 5.52E-24 |
| B4GALT7 | 2.9697 | 5.7881 | 0.9628 | 4.79E-27 | 3.98E-25 |
| B4GAT1 | 6.2598 | 12.0250 | 0.9418 | 1.00E-13 | 2.96E-13 |
| BBOX1 | 10.1848 | 5.3343 | -0.9330 | 4.39E-13 | 1.20E-12 |
| BCAT2 | 2.7089 | 6.3438 | 1.2276 | 2.26E-18 | 1.18E-17 |
| BCKDHB | 10.3186 | 6.2039 | -0.7340 | 6.18E-18 | 3.12E-17 |
| BHMT | 115.8564 | 50.9306 | -1.1857 | 4.92E-14 | 1.53E-13 |
| CAT | 102.3399 | 50.9365 | -1.0066 | 2.26E-14 | 7.20E-14 |
| CHPF | 12.0276 | 18.1183 | 0.5911 | 0.0050 | 0.0060 |
| CHPF2 | 4.2796 | 8.0051 | 0.9034 | 6.55E-19 | 3.69E-18 |
| CKB | 0.9746 | 15.0830 | 3.9519 | 4.13E-08 | 7.38E-08 |
| CPS1 | 119.0181 | 76.2365 | -0.6426 | 1.81E-07 | 3.15E-07 |
| CYB5R1 | 5.0407 | 12.4474 | 1.3042 | 1.52E-25 | 4.45E-24 |
| CYB5R3 | 17.4096 | 26.9935 | 0.6327 | 2.01E-07 | 3.47E-07 |
| CYP1A2 | 109.0277 | 15.6270 | -2.8026 | 5.98E-32 | 1.75E-29 |
| CYP1B1 | 2.1806 | 5.4174 | 1.3129 | 0.0069 | 0.0082 |
| DARS2 | 2.8442 | 6.8915 | 1.2768 | 1.21E-23 | 1.78E-22 |
| DBH | 9.2142 | 2.8538 | -1.6910 | 1.78E-24 | 3.26E-23 |
| DNMT1 | 2.8087 | 5.1255 | 0.8678 | 7.50E-21 | 5.70E-20 |
| DNMT3A | 2.1347 | 3.3191 | 0.6367 | 7.94E-24 | 1.29E-22 |
| DOT1L | 1.9703 | 3.0025 | 0.6077 | 7.39E-16 | 2.61E-15 |
| DPYS | 54.9850 | 32.8457 | -0.7433 | 2.34E-09 | 4.54E-09 |
| ECHS1 | 265.9524 | 148.5740 | -0.8400 | 5.40E-13 | 1.44E-12 |
| EHHADH | 42.9171 | 24.7097 | -0.7965 | 2.36E-10 | 5.15E-10 |
| EHMT2 | 2.5755 | 6.6626 | 1.3712 | 4.23E-28 | 6.20E-26 |
| ENOPH1 | 5.0851 | 9.4865 | 0.8996 | 2.78E-21 | 2.60E-20 |
| EXT1 | 7.1503 | 10.5084 | 0.5555 | 6.39E-05 | 8.83E-05 |
| FARS2 | 3.7813 | 6.1642 | 0.7050 | 5.19E-19 | 2.98E-18 |
| FARSA | 6.2584 | 13.1728 | 1.0737 | 4.85E-22 | 5.26E-21 |
| FTCD | 93.1913 | 46.4915 | -1.0032 | 6.10E-13 | 1.61E-12 |
| GALE | 7.2439 | 12.6667 | 0.8062 | 2.02E-11 | 4.84E-11 |
| GALK1 | 11.2010 | 23.5576 | 1.0726 | 9.53E-05 | 0.0001 |
| GATM | 116.2280 | 71.2458 | -0.7061 | 1.09E-08 | 2.05E-08 |
| GCDH | 17.4322 | 10.4407 | -0.7395 | 2.95E-12 | 7.32E-12 |
| GFPT1 | 4.7982 | 7.3422 | 0.6137 | 2.23E-11 | 5.27E-11 |
| GGT5 | 6.4136 | 4.2671 | -0.5879 | 1.15E-12 | 2.95E-12 |
| GLB1 | 7.1072 | 12.9931 | 0.8704 | 8.00E-18 | 3.97E-17 |
| GLS | 2.4083 | 4.5066 | 0.9040 | 3.87E-12 | 9.44E-12 |
| GLS2 | 7.0650 | 4.5709 | -0.6282 | 1.24E-15 | 4.29E-15 |
| GLUD1 | 101.7168 | 69.2661 | -0.5543 | 8.26E-10 | 1.66E-09 |
| GMDS | 3.5869 | 6.4448 | 0.8454 | 3.91E-10 | 8.30E-10 |
| GMPPA | 4.2410 | 8.4722 | 0.9983 | 1.45E-24 | 2.84E-23 |
| GMPPB | 2.8940 | 4.5698 | 0.6591 | 8.29E-23 | 1.10E-21 |
| GNE | 18.5082 | 10.0160 | -0.8859 | 2.36E-17 | 1.05E-16 |
| GNMT | 81.9484 | 40.5399 | -1.0154 | 4.62E-11 | 1.07E-10 |
| GNPDA1 | 3.3310 | 6.5654 | 0.9789 | 3.86E-20 | 2.51E-19 |
| GNS | 7.3114 | 17.3480 | 1.2465 | 3.56E-19 | 2.09E-18 |
| GOT1 | 88.0512 | 59.8293 | -0.5575 | 1.04E-06 | 1.68E-06 |
| GOT2 | 51.6621 | 36.1547 | -0.5149 | 2.46E-09 | 4.75E-09 |
| GPI | 10.5810 | 25.6124 | 1.2754 | 1.93E-16 | 7.56E-16 |
| GSTZ1 | 11.0418 | 5.4838 | -1.0097 | 2.84E-21 | 2.60E-20 |
| HAAO | 49.2764 | 25.0653 | -0.9752 | 3.59E-12 | 8.84E-12 |
| HARS2 | 3.5891 | 6.8555 | 0.9336 | 8.15E-27 | 3.98E-25 |
| HEXA | 4.1802 | 6.6626 | 0.6725 | 1.23E-18 | 6.66E-18 |
| HEXB | 9.0906 | 20.0698 | 1.1426 | 3.86E-21 | 3.42E-20 |
| HMGCL | 27.2543 | 16.0771 | -0.7615 | 2.52E-16 | 9.60E-16 |
| HMGCS1 | 13.7162 | 24.4448 | 0.8336 | 0.0001 | 0.0001 |
| HMGCS2 | 373.4068 | 220.9075 | -0.7573 | 5.51E-10 | 1.15E-09 |
| HS3ST3B1 | 7.6188 | 4.2256 | -0.8504 | 5.05E-14 | 1.56E-13 |
| HS6ST1 | 3.7345 | 8.4655 | 1.1807 | 6.11E-10 | 1.25E-09 |
| HYAL2 | 4.7522 | 8.0524 | 0.7608 | 3.14E-17 | 1.37E-16 |
| HYAL3 | 2.4382 | 3.7295 | 0.6132 | 1.01E-06 | 1.65E-06 |
| IARS2 | 10.9000 | 21.1593 | 0.9570 | 6.27E-14 | 1.87E-13 |
| IDUA | 2.0216 | 4.2545 | 1.0735 | 1.02E-25 | 3.31E-24 |
| IVD | 21.6424 | 12.9903 | -0.7364 | 6.68E-16 | 2.39E-15 |
| KMO | 8.6095 | 5.0044 | -0.7827 | 8.62E-18 | 4.21E-17 |
| LCMT1 | 3.2976 | 5.7772 | 0.8089 | 5.58E-25 | 1.36E-23 |
| MAT1A | 298.9184 | 103.4346 | -1.5310 | 1.99E-21 | 2.00E-20 |
| MAT2A | 7.6682 | 11.8293 | 0.6254 | 0.0001 | 0.0001 |
| MIF | 8.9528 | 28.0273 | 1.6464 | 2.49E-13 | 7.15E-13 |
| MTR | 3.2704 | 4.7977 | 0.5529 | 2.77E-16 | 1.03E-15 |
| NANS | 5.0062 | 7.5206 | 0.5871 | 7.18E-17 | 2.96E-16 |
| NDST1 | 3.5061 | 9.2511 | 1.3998 | 1.44E-20 | 9.79E-20 |
| NPL | 3.2828 | 4.9077 | 0.5801 | 0.0126 | 0.0146 |
| NSD1 | 2.4891 | 3.6327 | 0.5454 | 1.27E-20 | 9.05E-20 |
| ODC1 | 10.1526 | 20.7547 | 1.0316 | 1.65E-08 | 2.99E-08 |
| OGDH | 7.7673 | 15.6264 | 1.0085 | 4.59E-13 | 1.25E-12 |
| OGDHL | 19.0076 | 10.5346 | -0.8514 | 4.39E-13 | 1.20E-12 |
| OTC | 52.8794 | 33.6998 | -0.6500 | 6.06E-07 | 1.01E-06 |
| OXCT1 | 1.9554 | 2.8264 | 0.5315 | 0.0002 | 0.0003 |
| P4HA2 | 2.6032 | 5.0393 | 0.9529 | 1.64E-22 | 2.00E-21 |
| PAPSS1 | 3.6193 | 5.9114 | 0.7078 | 7.56E-17 | 3.03E-16 |
| PDHA1 | 9.6956 | 13.7331 | 0.5022 | 7.54E-11 | 1.70E-10 |
| PGM1 | 37.7408 | 23.0740 | -0.7099 | 1.11E-13 | 3.25E-13 |
| PHGDH | 16.3186 | 9.2302 | -0.8221 | 2.77E-11 | 6.44E-11 |
| PLOD1 | 10.9464 | 26.3683 | 1.2683 | 1.02E-14 | 3.31E-14 |
| PLOD2 | 6.7924 | 9.7384 | 0.5197 | 0.0018 | 0.0023 |
| PLOD3 | 4.5372 | 15.8417 | 1.8038 | 6.33E-27 | 3.98E-25 |
| PRDX6 | 79.8923 | 114.7817 | 0.5228 | 0.0014 | 0.0017 |
| PRODH2 | 24.9677 | 17.4394 | -0.5177 | 1.11E-07 | 1.96E-07 |
| PSAT1 | 35.9448 | 21.3248 | -0.7533 | 2.96E-10 | 6.37E-10 |
| PSPH | 2.6549 | 7.6440 | 1.5257 | 3.94E-24 | 6.79E-23 |
| PYCR1 | 1.3413 | 6.4395 | 2.2633 | 9.35E-06 | 1.41E-05 |
| RARS2 | 4.0516 | 6.2752 | 0.6311 | 2.04E-09 | 3.98E-09 |
| SARDH | 22.0066 | 10.8674 | -1.0179 | 1.74E-17 | 7.98E-17 |
| SEPHS1 | 5.6419 | 9.7620 | 0.7910 | 1.75E-20 | 1.16E-19 |
| SEPHS2 | 56.0266 | 99.8468 | 0.8336 | 2.59E-10 | 5.62E-10 |
| SETD1A | 2.8444 | 4.2936 | 0.5941 | 2.04E-21 | 2.00E-20 |
| SETDB1 | 2.6809 | 4.8658 | 0.8599 | 6.34E-25 | 1.43E-23 |
| SGSH | 3.2901 | 4.7396 | 0.5267 | 4.76E-21 | 3.99E-20 |
| SHMT1 | 32.2637 | 18.0428 | -0.8385 | 1.42E-12 | 3.58E-12 |
| SMS | 6.6735 | 13.2909 | 0.9939 | 7.48E-17 | 3.03E-16 |
| SRM | 5.3783 | 19.6411 | 1.8687 | 2.34E-23 | 3.26E-22 |
| SUV39H1 | 2.7018 | 4.9637 | 0.8775 | 7.15E-26 | 2.62E-24 |
| SUV39H2 | 1.6782 | 2.5312 | 0.5928 | 8.29E-25 | 1.74E-23 |
| TARS2 | 5.0885 | 8.9261 | 0.8108 | 1.31E-16 | 5.18E-16 |
| TAT | 154.1952 | 87.1227 | -0.8236 | 6.25E-10 | 1.27E-09 |
| TDO2 | 56.5127 | 22.2686 | -1.3436 | 1.23E-15 | 4.29E-15 |
| TSTA3 | 6.5225 | 16.9466 | 1.3775 | 9.59E-18 | 4.61E-17 |
| UGDH | 12.4510 | 25.3022 | 1.0230 | 0.0002 | 0.0003 |
| UGP2 | 37.3764 | 24.4160 | -0.6143 | 1.31E-10 | 2.93E-10 |
| UXS1 | 3.2357 | 5.5719 | 0.7841 | 1.67E-26 | 6.98E-25 |
| XYLT2 | 2.6953 | 5.0655 | 0.9103 | 1.03E-23 | 1.59E-22 |

Abbreviations: FC, fold change; fdr, false discovery rate.

**Table**.**S5** Amino acid metabolism-related prognostic genes by univariate COX analyzing

| Gene name | HR | 95%CI | p-value |
| --- | --- | --- | --- |
| ABAT | 0.9815 | 0.9684 - 0.9949 | 0.0070 |
| ACAT1 | 0.9819 | 0.9710 - 0.9928 | 0.0012 |
| ADH1A | 0.9965 | 0.9943 - 0.9987 | 0.0020 |
| ADH6 | 0.9869 | 0.9787 - 0.9952 | 0.0019 |
| ALAS1 | 0.9926 | 0.9885 - 0.9968 | 0.0005 |
| ALDH2 | 0.9885 | 0.9814 - 0.9956 | 0.0015 |
| B3GAT3 | 1.0372 | 1.0176 - 1.0571 | 0.0002 |
| B4GALT2 | 1.0875 | 1.0488 - 1.1276 | 5.56E-06 |
| B4GALT3 | 1.1042 | 1.0500 - 1.1612 | 0.0001 |
| CHPF2 | 1.0919 | 1.0444 - 1.1416 | 0.0001 |
| CYB5R3 | 1.0212 | 1.0107 - 1.0320 | 7.74E-05 |
| DARS2 | 1.0724 | 1.0290 - 1.1177 | 0.0009 |
| DNMT1 | 1.1123 | 1.0591 - 1.1682 | 2.11E-05 |
| DNMT3A | 1.3472 | 1.1801 - 1.5379 | 1.03E-05 |
| DOT1L | 1.3159 | 1.1330 - 1.5283 | 0.0003 |
| DPYS | 0.9899 | 0.9832 - 0.9966 | 0.0032 |
| EHMT2 | 1.0728 | 1.0198 - 1.1285 | 0.0065 |
| ENOPH1 | 1.0852 | 1.0375 - 1.1352 | 0.0004 |
| GATM | 0.9959 | 0.9930 - 0.9989 | 0.0069 |
| GLS | 1.0999 | 1.0434 - 1.1595 | 0.0004 |
| GMPPA | 1.0656 | 1.0191 - 1.1142 | 0.0052 |
| GNPDA1 | 1.1025 | 1.0622 - 1.1443 | 2.80E-07 |
| GOT2 | 0.9821 | 0.9732 - 0.9910 | 9.37E-05 |
| HARS2 | 1.1388 | 1.0636 - 1.2193 | 0.0002 |
| HEXB | 1.0427 | 1.0238 - 1.0620 | 7.20E-06 |
| HMGCS2 | 0.9984 | 0.9974 - 0.9994 | 0.0014 |
| IVD | 0.9547 | 0.9284 - 0.9817 | 0.0011 |
| NPL | 1.0442 | 1.0142 - 1.0749 | 0.0036 |
| NSD1 | 1.3173 | 1.1115 - 1.5613 | 0.0015 |
| OTC | 0.9919 | 0.9859 - 0.9979 | 0.0079 |
| PAPSS1 | 1.0950 | 1.0370 - 1.1562 | 0.0011 |
| PLOD1 | 1.0150 | 1.0075 - 1.0225 | 8.41E-05 |
| PLOD2 | 1.0669 | 1.0448 - 1.0895 | 1.38E-09 |
| PSPH | 1.0424 | 1.0164 - 1.0689 | 0.0012 |
| PYCR1 | 1.0275 | 1.0107 - 1.0446 | 0.0013 |
| SEPHS1 | 1.1326 | 1.0925 - 1.1743 | 1.37E-11 |
| SMS | 1.0398 | 1.0254 - 1.0544 | 4.21E-08 |
| SRM | 1.0161 | 1.0077 - 1.0247 | 0.0002 |
| SUV39H2 | 1.7337 | 1.4286 - 2.1039 | 2.52E-08 |
| UGDH | 1.0120 | 1.0056 - 1.0184 | 0.0002 |
| UXS1 | 1.1366 | 1.0382 - 1.2442 | 0.0056 |
